# Breastfeeding in Women with Postpartum Depression: A Systematic Review and Meta-Analysis

**DOI:** 10.4314/ejhs.v35i4.10

**Published:** 2025-07

**Authors:** Maryam Zamani, Rasoul Alimi, Sedigheh Abdollahpour, Elham Azmoude

**Affiliations:** 1 Department of Midwifery, Neyshabur University of Medical Sciences, Neyshabur, Iran; 2 Department of Epidemiology and Biostatistics, School of Health, Torbat Heydariyeh University of Medical Sciences, Torbat Heydariyeh, Iran; 3 Nursing and Midwifery Care Research Center, Mashhad University of Medical Sciences, Mashhad, Iran; 4 Department of Midwifery, School of Nursing and Midwifery, Neyshabur University of Medical Sciences, Neyshabur, Iran

**Keywords:** Postpartum Depression, Post-natal Depression, Breastfeeding, Exclusive Breastfeeding, Lactation

## Abstract

**Background:**

Postpartum depression has been associated with difficulties in initiating and sustaining breastfeeding. While several observational studies have explored this relationship, their findings remain inconsistent, and comprehensive meta-analyses are limited. This study aimed to evaluate breastfeeding practices among women experiencing postpartum depression.

**Methods:**

A systematic literature search was conducted in accordance with the Preferred Reporting Items for Systematic Reviews and Meta-Analyses (PRISMA) guidelines, covering publications from inception through April 2023. Databases searched included MEDLINE via PubMed, Scopus, Web of Science, and Google Scholar. Search terms included both medical subject headings and relevant keywords: “Breast Feeding,” “Breast Milk Expression,” “Exclusive Breastfeeding,” “Breastfeeding,” “Postnatal Depression,” “Postpartum Depression,” “Puerperal Depression,” “Lactation,” “Human Milk,” and “Breast Milk.” Only studies utilizing the Edinburgh Postnatal Depression Scale (EPDS) were included. The quality of the included studies was assessed using the Strengthening the Reporting of Observational Studies in Epidemiology (STROBE) checklist.

**Results:**

Of the 1,514 articles screened, nine met the eligibility criteria. The findings indicate that women with postpartum depression had significantly higher odds of practicing nonexclusive breastfeeding compared to those without depression (OR = 2.25; 95% CI: 1.46–3.45; P < .001). A subgroup analysis of studies using an EPDS cut-off score of ≥12 revealed that non-exclusive breastfeeding was 71% more likely among women with depressive symptoms (OR = 1.71; 95% CI: 1.40–2.08).

**Conclusions:**

Postpartum depressive symptoms are significantly associated with an increased risk of discontinuing exclusive breastfeeding. These findings underscore the importance of integrating mental health screening and breastfeeding support into routine postpartum care to identify at-risk women and implement timely interventions that can enhance both maternal and neonatal outcomes.

## Introduction

The postpartum period is widely recognized as one of the most challenging phases in a woman's life, during which many experience cognitive and mood disturbances (1). Postpartum depression (PPD), a prevalent mood disorder during this time, affects approximately 17% of otherwise healthy women and is characterized by persistent sadness, low mood, chronic fatigue, and a lack of energy (2,3).

PPD imposes a significant burden not only on the affected mothers but also on their infants, partners, and other family members. It often leads to impaired social functioning and strained marital relationships (4,5). Furthermore, PPD may disrupt the mother-infant bond, potentially delaying the infant's cognitive and emotional development (6).

Among the many maternal behaviors impacted by mental health, breastfeeding is particularly susceptible. Breastfeeding is widely recommended due to its well-established health benefits for both the mother and infant (7,8). However, depressive symptoms may negatively affect breastfeeding practices through various mechanisms, such as diminished maternal self-efficacy, emotional withdrawal, fatigue, and difficulty establishing physical closeness with the infant (9,10).

Although numerous observational studies have explored the relationship between PPD and breastfeeding, their findings remain inconsistent. The absence of a comprehensive and current metaanalysis further obscures the evidence base (11–16). For instance, a meta-analysis focusing solely on African women found that PPD significantly impacted exclusive breastfeeding rates (17). In contrast, an observational study by Fukui et al. reported no bidirectional relationship between depression and breastfeeding practices at four weeks postpartum (18).

This study aimed to systematically review the existing literature to evaluate breastfeeding behaviors among women with postpartum depression. The findings are intended to guide clinicians, researchers, and policymakers by clarifying the extent to which PPD affects breastfeeding, thereby supporting the development of integrated interventions that address both maternal mental health and infant nutrition.

## Materials and Methods

This review followed the Preferred Reporting Items for Systematic Reviews and Meta-Analyses (PRISMA) guidelines (19). The systematic review protocol was registered with the International Prospective Register of Systematic Reviews (CRD42021290984).

**Search strategy**: An extensive, systematic search was conducted to identify studies examining breastfeeding among women diagnosed with PPD. Searches were carried out in English across multiple electronic databases—PubMed, Scopus, Web of Science, and Google Scholar—from their inception through April 2023. Both Medical Subject Headings (MeSH) and non-MeSH terms were used, including: “Breast Feeding,” “Breast Milk Expression,” “Exclusive Breastfeeding,” “Feeding,” “Post Natal Depression,” “Post-Natal Depression,” “Post-Partum Depression,” “Postnatal Depression,” “Puerperal Depression,” “Postpartum,” “Exclusive Breast Feeding,” “Lactation,” “Human Milk,” and “Breast Milk.” Advanced search techniques were applied, such as using quotation marks for exact phrases, parentheses for grouping terms, and asterisks for capturing term variations. Reference lists of selected studies were also screened to identify additional relevant publications.

All retrieved citations were imported into EndNote software (version 20). After removing duplicates, two reviewers (MZ and NM) independently screened titles and abstracts to identify eligible studies. Discrepancies or uncertainties were resolved through consultation with a third reviewer (RA). No publication date restrictions were applied.

**Inclusion and exclusion criteria**: Inclusion criteria were established using an adapted PICOS framework (20) and consisted of the following: (a) studies evaluating the association between PPD and breastfeeding outcomes (exclusive vs. nonexclusive) within one week to one year postpartum—based on evidence supporting the predictive validity of the Edinburgh Postnatal Depression Scale (EPDS) during this period; (b) use of the EPDS or another validated tool to assess depression; (c) retrospective or prospective study design; (d) sample size greater than 25 to ensure statistical power; and (e) studies providing point estimates (e.g., odds ratios [ORs]) comparing breastfeeding outcomes between women with and without PPD or sufficient data to calculate them (19).

**Figure 1:**
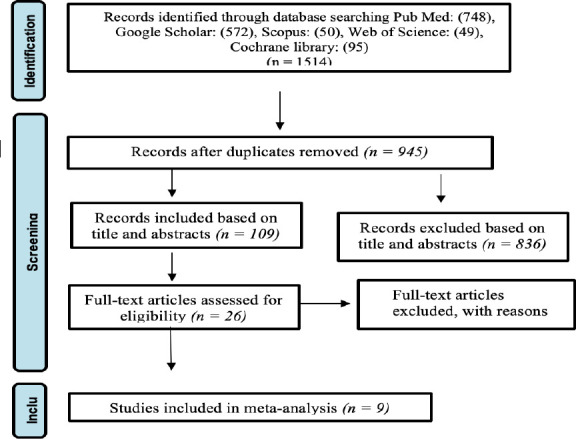
PRISMA flow diagram

**Exclusion criteria**: (a) studies not published in English; (b) lack of access to full-text articles or insufficient data in abstracts; (c) data inconsistencies; and (d) use of inappropriate statistical methods. Following this selection process, 10 full-text articles were deemed eligible for inclusion and underwent further assessment (Figure 1).

**Data extraction and study quality assessment**: Data were extracted using a pre-designed Excel form developed for this review. The following details were recorded for each study: first author's last name, year of publication, country, sample size, study duration, participant age (mean or range), sample sizes for exclusive breastfeeding and the comparison group (non-exclusive or no breastfeeding) and point estimates comparing breastfeeding outcomes between women with and without postpartum depression.

In instances where studies reported the inverse comparison—i.e., breastfeeding outcomes among non-depressed versus depressed participants—odds ratios (ORs) were mathematically inverted to ensure consistency in interpretation across studies. These inverted estimates are indicated with an asterisk in the data table. Where available, the reported prevalence of PPD was also extracted and is summarized in Table 1.

**Table 1:** The baseline features of the systematic reviews and meta-analysis studies on the postpartum depression effect on the type of breastfeeding

Study author's	publication year	country	Sample size	Age Mean ± SD	time duration	Effect size		EPDS cut point	Prevalence of postpartum depression	Main Concepts

						OR (95%CI)				
Dennis [22]	2007	Canada	594	28.50±5.00	4 weeks	1.64	(0.94-2.86) *	12	14.6	Early PPD linked to lower breastfeeding continuation
Dennis [22]	2007	Canada	594	28.50±5.00	8 weeks	1.75	(1.05-2.92) *	12	14.6	
Nishioka [23]	2011	Japan	405	Not reported	5 months	2.93	(1.26-6.78)	9	13.0	Increased risk of breastfeeding disruption associated with postpartum depression
Madeghe [24]	2016	Kenya	200	17-39 (range)	6-16 weeks	5.91	(2.29-15.27)	13	13.0	Postpartum depression strongly associated with non-exclusive breastfeeding
Imširagić [25]	2016	Croatia	259	Not reported	6-9 weeks	1.09	(1.01-1.18) *	9-14	Not reported	Higher postpartum depression scores were associated with lower odds of exclusive breastfeeding
Islam [26]	2016	Bangladesh	426	Not reported	6 months	5.0	(2.27-11.01) *	10	35.2	Postpartum depression is significantly associated with a lower likelihood of exclusive breastfeeding.
Silva [27]	2016	Brazil	2583	Not reported	3 months	1.63	(1.20-2.20)	12	11.8	PPD increased the likelihood of discontinuing exclusive breastfeeding.
Woolhouse [28]	2016	Australia	1507	Not reported	6 months	1.82	(1.11-2.98) *	13	Not reported	Depressive symptoms at three months postpartum reduced breastfeeding odds at six months
Goyal [10]	2017	India	479	Not reported	1 week	12.50	(4.01-38.97) *	11	11.9	High PPD scores were associated with lower odds of exclusive breastfeeding.
Ezzeddin [11]	32019	Iran	325	28.62±5.67	3-8 months	1.10	(0.59-2.04)	13	35.4	PPD was significantly associated with lower odds of exclusive breastfeeding.

**Methodological quality assessment and statistical analysis**: The methodological quality of the included studies was assessed using the Strengthening the Reporting of Observational Studies in Epidemiology (STROBE) checklist (21).

Pooled estimates were calculated using odds ratios (ORs) to evaluate the association between postpartum depression (PPD) and breastfeeding type across all included studies. When available, adjusted ORs were prioritized. Each OR was presented with a corresponding 95% confidence interval (CI). Forest plots were used to illustrate individual study findings, pooled effect estimates, and the extent of heterogeneity.

A random-effects model was applied to account for the assumption that the included studies represent a random sample from a broader population. Heterogeneity was quantified using the I^2^ statistic, with thresholds of 25%, 50%, and 75% interpreted as low, moderate, and high heterogeneity, respectively (22). To investigate potential sources of heterogeneity, subgroup analyses were conducted based on the Edinburgh Postnatal Depression Scale (EPDS) cut-off scores (<12 vs. ≥12), as reported in the included studies.

All statistical analyses were conducted using Stata version 17.0 (College Station, TX: StataCorp LLC), with statistical significance defined as p < 0.05.

## Results

Out of 1,514 studies identified through the literature search, nine studies—reporting a total of ten effect sizes—met the eligibility criteria and were included in the systematic review and metaanalysis (11,12,23–29). These studies collectively involved 6,778 participants, with follow-up periods ranging from one week to eight months. The included studies were published between 2007 and 2019, as summarized in Table 1.

**Figure 2:**
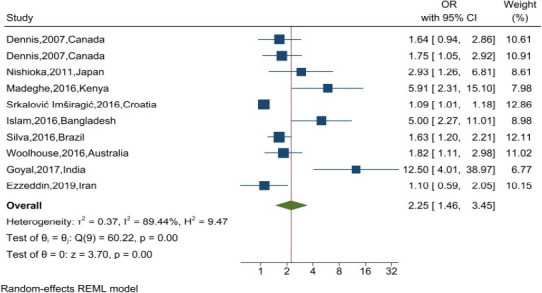
The forest plot diagram of the systematic reviews and meta-analysis studies showing the effect of postnatal depression on exclusive breastfeeding

The association between PPD and breastfeeding type is illustrated in Figure 2. The pooled odds ratio for nonexclusive breastfeeding among women with PPD was 2.25 (95% CI: 1.46–3.45; p < .001), indicating that women with PPD had more than twice the odds of practicing nonexclusive breastfeeding compared to those without PPD. Substantial heterogeneity was observed among the included studies (I^2^ = 89.44%, Q test: p < .001).

**Figure 3:**
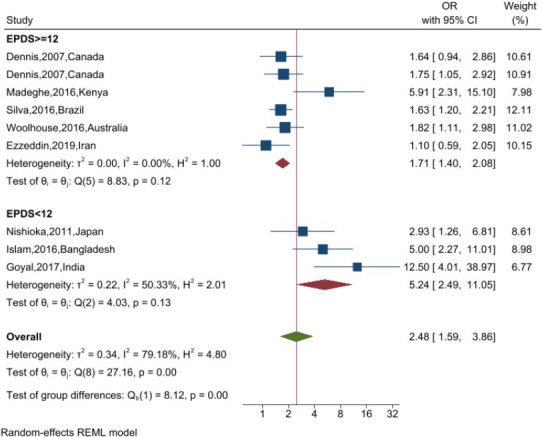
The subgroup analysis for the postpartum depression effect on the type of breastfeeding

Subgroup analyses based on EPDS cut-off scores are presented in Figure 3. Nine studies, reporting a total of ten effect sizes, evaluated the association between maternal depression and non-exclusive breastfeeding. The random-effects meta-analysis showed that in studies using an EPDS cut-off score of ≥12 to define depressive symptoms, depressed mothers were 71% more likely to practice non-exclusive breastfeeding (OR = 1.71; 95% CI: 1.40–2.08). In contrast, studies using a cut-off score of <12 reported a substantially stronger association, with depressed mothers nearly five times more likely to engage in non-exclusive breastfeeding (OR = 5.24; 95% CI: 2.49–11.05). The difference in effect sizes between these two subgroups was statistically significant (p < 0.001).

Regarding heterogeneity, a moderate level was observed among studies using an EPDS cut-off of <12, whereas no heterogeneity was detected in studies using a cut-off of ≥12.

**Figure 4:**
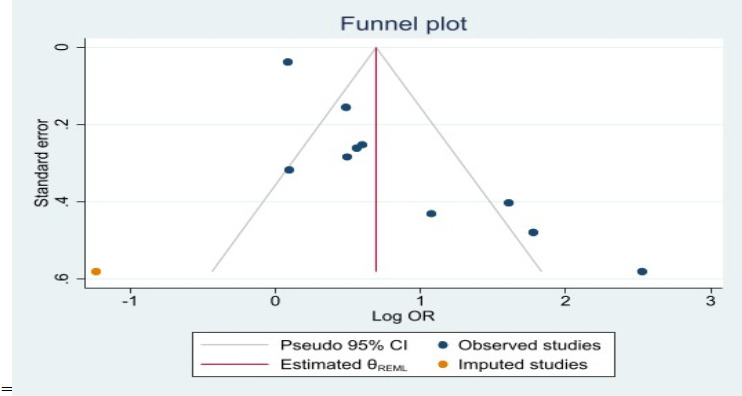
The funnel plot for checking publication bias

**Publication bias**: The funnel plot suggests a publication bias (Figure 4), which further supported by Egger's test (the bias coefficients b) 3.18; z = 4.67, P < 0.001). These results indicate that publication bias may significantly affect the overall estimate. To account for this, the Duval and Tweedie non-parametric trim-and-fill method was applied. According to this analysis, one study was imputed and removed from the funnel plot, resulting in an adjusted effect size of OR = 2.01, 95% CI (1.22, 3.31), slightly lower than the original unadjusted OR of 2.25.

**Figure 5:**
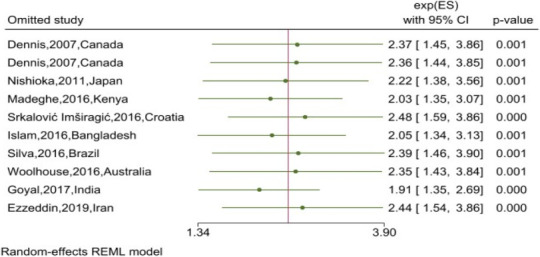
Assessing the influence of a single study on the pooled estimate of the postnatal depression effect on the type of breastfeeding

**Sensitivity tests**: The sensitivity tests indicated that all the single-study omitted estimates laid within the 95% CI of the respective overall effect. This suggested that the pooled effect was not substantially influenced by any single study. The stability of such results validated the rationality and reliability of our analyses (Figure 5).

## Discussion

Our findings indicate that women experiencing postpartum depression (PPD) have nearly twice the odds of practicing non-exclusive breastfeeding compared to those without depressive symptoms during the first six months postpartum. This result is consistent with previous research. Multiple studies have reported associations between PPD and negative attitudes toward breastfeeding, decreased satisfaction with the breastfeeding experience, greater difficulty initiating breastfeeding, and earlier cessation (30–32).

For example, Wouk et al. (2017) found that PPD and anxiety were associated with lower rates of breastfeeding initiation, shorter breastfeeding duration, and reduced intensity (33). The mechanisms underlying this association are likely multifactorial. Women with PPD often report diminished confidence in their maternal role, reduced breastfeeding self-efficacy, more negative interactions with their infants, and a decreased perception of breastfeeding benefits. Additionally, neuroendocrine disruptions—such as altered oxytocin and prolactin regulation—have been observed in mothers with depression and may directly impair lactation (30–32). These findings underscore the importance of addressing maternal mental health as a key factor influencing exclusive breastfeeding.

However, some studies have found no significant association between PPD and breastfeeding outcomes (12,34). Notably, a metaanalysis of studies conducted in sub-Saharan Africa reported no significant effect of PPD on exclusive breastfeeding (16). This inconsistency may reflect contextual differences, such as sociodemographic and socioeconomic factors unique to sub-Saharan African countries, as well as variability in the tools used to assess PPD.

Further complexity is introduced by findings such as those from Misri et al., who reported that while the onset of PPD accelerated breastfeeding cessation, the severity of depressive symptoms did not significantly influence breastfeeding duration (35). Similarly, Henderson et al. (2003) observed that over 80% of Australian women discontinued breastfeeding following the onset of depression, with approximately 10% doing so immediately after diagnosis (30). Other studies suggest that even antenatal depression may negatively influence breastfeeding outcomes (36).

Several studies propose that the relationship between PPD and exclusive breastfeeding may be bidirectional and complex (31,32). For instance, Gagliardi et al. (2009) found that women with more pronounced early postpartum depressive symptoms were more likely to switch to bottle feeding by three months postpartum (37). These findings support a robust association between the onset of PPD and breastfeeding challenges. Future research should stratify depressive symptoms by severity to determine whether mild, moderate, or severe depression differentially affects breastfeeding behavior.

In conclusion, this meta-analysis suggests that women with PPD are at significantly increased risk of discontinuing exclusive breastfeeding. Therefore, comprehensive postpartum care should include both mental health screening and breastfeeding support, particularly in the early postpartum period. Integrating mental health assessments into routine postpartum care can facilitate early identification of at-risk women and enable timely, targeted interventions. The early detection and treatment of PPD—alongside breastfeeding counseling—may improve both maternal and neonatal health outcomes.

Further research is warranted to examine how the severity of depressive symptoms influences breastfeeding patterns and to evaluate the effectiveness of early interventions. Identifying mediating factors—such as social support, healthcare access, and cultural practices—will be essential in designing effective public health strategies to support breastfeeding among women with PPD. Culturally sensitive, multidisciplinary approaches will be particularly important for improving breastfeeding outcomes on a global scale.

This study has several strengths. The included studies were conducted across a range of countries, enhancing the generalizability of the findings. In addition, the robustness of the results is supported by sensitivity analyses, which indicated that no single study unduly influenced the overall estimates.

Nonetheless, these findings should be interpreted with caution. The cross-sectional design of most included studies limits the ability to infer causality between PPD and breastfeeding practices. Moreover, the observed heterogeneity among studies—particularly in relation to the cut-off scores used for depression screening—may have influenced the pooled estimates.
